# AI-augmented ECG for pre-echocardiography triage: a tool to optimize cardiac imaging utilization

**DOI:** 10.1093/ehjdh/ztag082

**Published:** 2026-06-12

**Authors:** Abhyuday Kumara Swamy, Deepak Krishnan, Pranay Narhari Umredkar, Aditya HN, Santhosh Rathnam Palani, Vivek Rajagopal, Pradeep Narayan, Deepak Padmanabhan

**Affiliations:** Medha AI, Narayana Health, 3rd Floor, Hustle Hub, No.8, 17th crossroad, 7th sector, HSR Layout, 560102 Bengaluru, Karnataka, India; Medha AI, Narayana Health, 3rd Floor, Hustle Hub, No.8, 17th crossroad, 7th sector, HSR Layout, 560102 Bengaluru, Karnataka, India; Medha AI, Narayana Health, 3rd Floor, Hustle Hub, No.8, 17th crossroad, 7th sector, HSR Layout, 560102 Bengaluru, Karnataka, India; Medha AI, Narayana Health, 3rd Floor, Hustle Hub, No.8, 17th crossroad, 7th sector, HSR Layout, 560102 Bengaluru, Karnataka, India; Medha AI, Narayana Health, 3rd Floor, Hustle Hub, No.8, 17th crossroad, 7th sector, HSR Layout, 560102 Bengaluru, Karnataka, India; Medha AI, Narayana Health, 3rd Floor, Hustle Hub, No.8, 17th crossroad, 7th sector, HSR Layout, 560102 Bengaluru, Karnataka, India; Department of Cardio-Thoracic Surgery, Rabindranath Tagore International Institute of Cardiac Sciences, Narayana Health, Kolkata, India; Department of Electrophysiology, Narayana Institute of Cardiac Sciences, Narayana Health, Bengaluru, India

**Keywords:** ECG-AI, Echocardiography, Artificial intelligence, Cardiac screening

## Abstract

**Aims:**

Echocardiography is a key diagnostic modality for cardiac dysfunction but is often over utilized due to variability in pre-test clinical assessment. There is a need for a scalable, cost-effective screening tool that can reduce unnecessary referrals without compromising diagnostic accuracy. To develop and validate an AI tool that uses standard 12-lead ECG images to predict the presence of major echocardiographic abnormalities, including reduced ejection fraction (EF ≤35%), valvular heart disease, and elevated pulmonary artery pressure, as a triage tool prior to echocardiography.

**Methods and results:**

51,055 patients aged ≥15 years from a tertiary cardiac care centre, which underwent ECG and echocardiography on the same day between January 2021, and February 2024 were identified. ECGs were stored as images and pre-processed for model input. Echocardiographic findings were extracted using structured reports and regular expression-based keyword searches. The final dataset (*n* = 52,817) was split into training (40,796), epoch-monitoring (2,148), and testing (9,873) sets. An ensemble of 3 deep learning models was trained. Model performance was assessed using AUROC, PRAUC, sensitivity, specificity, positive predictive value, and negative predictive value. The internal test set demonstrated an AUROC of 0.87 (95% CI: 0.86–0.88) and PRAUC of 0.66 (95% CI: 0.65–0.69). At the Youden threshold (0.27), sensitivity, specificity, PPV, and NPV were 0.80, 0.80, 0.46, and 0.95, respectively. External validation was performed on 20,053 patients. It yielded an AUROC of 0.84 and PRAUC of 0.50.

**Conclusion:**

The proposed AI model accurately identifies major echocardiographic abnormalities from ECG images, achieving high NPV and demonstrating strong generalizability.

## Graphical Abstract

**Graphical Abstract:**
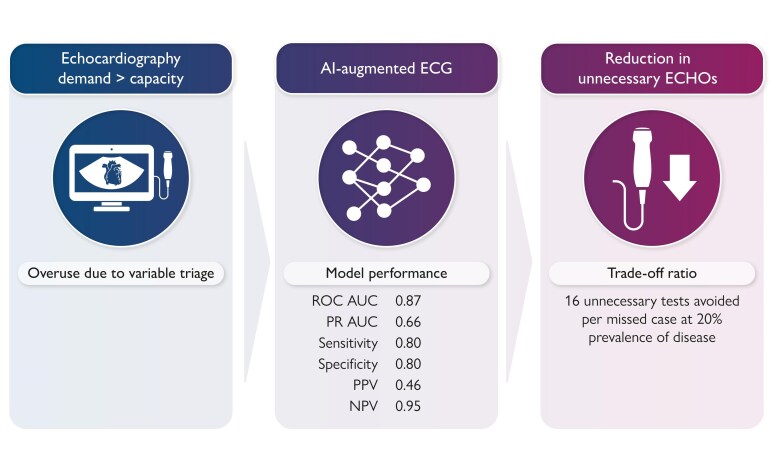

## Introduction

An echocardiogram is a valuable investigation in patients with suspected cardiac disease. However, the utility of the test is dependent on the pre-test probability of the abnormal cardiac function in the patient being referred to the echocardiogram lab.^1–3^ This depends on various factors including a detailed history and physical examination and interpretation of additional investigations like the electrocardiogram (ECG) and chest radiogram prior to referral for the echocardiogram.^3–5^ Variability in the skill levels and experience of the patient-facing health personnel may result in perhaps a higher-than-needed referral for an echocardiogram. This may result in higher patient health costs as well as higher load on the health care delivery systems thereby creating cascading downstream effects on quality and turnaround time for results.^6^ Moreover, both in India and in the western world, there seems to be an increase in the number of echocardiograms performed. The appropriateness of these echocardiograms, for detection of disease may not always be present, therefore, in a speciality resource limited, overburdened tertiary care system like India, a possible tool to aid referral of echocardiograms is valuable.^7–11^

Suspicion of abnormal cardiac function based on patient interaction and examination is also challenging for healthcare personnel who are not evaluating these patients daily.^12^ The cost of error in the detection of cardiac dysfunction is high.^13^ Therefore, there is a need for a screening tool to alert them to the likely possibility of cardiac disease. Additionally, busy outpatient clinics and low health care personnel-to-patient ratio necessitate rapid screening for cardiac dysfunction using modalities that are cheap, scalable, and need minimum time for their output results. The ideal tool is likely to have all the above in addition to being easily integrated into the existing workflow for patient management.

The ECG has been used for a century for the recording of cardiac electrical signals from the body surface based on clinically well-established and researched recording locations. It remains well entrenched in the workflow of cardiac care with the recording devices and the skill to record an ECG easily available and acceptable by the patient.

We aim to use the ubiquitous 12 lead ECG and aid healthcare personnel in screening and triaging the patients for referral to the echocardiogram lab by creating a novel deep learning algorithm designed to detect either one of low ejection fraction (EF), valvular heart disease (VHD) and elevated pulmonary artery pressure.

## Methodology

### Data and study population

#### Study sites

Model development was performed using data from hospitals located within the Narayana Health City campus in Bangalore, India, including the Narayana Institute of Cardiac Sciences and the Mazumdar Shaw Medical Centre. Narayana Institute of Cardiac Sciences is a Joint Commission International (JCI) and NABH-accredited tertiary cardiac centre and one of the flagship hospitals of the Narayana Health network, equipped with multiple cardiac operating theatres, catheterization laboratories, and specialized critical care units for adult and paediatric cardiac care. The NH Health City campus functions as a large tertiary referral hub providing advanced cardiovascular and multispecialty care with a high procedural volume.

Multi-site validation was conducted using data from the Rabindranath Tagore International Institute of Cardiac Sciences in Kolkata, India (hereafter referred to as the external site with an external validation cohort/dataset). This institution is a 681-bed NABH-accredited multi-super speciality tertiary and quaternary care hospital within the Narayana Health network, providing advanced cardiac and multispecialty services to patients across Eastern and Northeastern India as well as neighbouring countries.

### Data acquisition and compute specifications

Data was collected from Narayana Health, Bommasandra, Bengaluru retrospectively from electronic medical records (EMR) of the cardiac outpatient clinic between January 2021 to February 2024. All patients included in the database for this study had consented to the use of their data for research purposes. Approval for the study was obtained from the Institutional Review Board (IRB) within the institute. All patients 15 years and above were included in the study. The dataset for the study was used by selecting retrospectively from the EMR in the above time-period, patients having echo and ECG within the same day of each other. In case of more than one of each investigation, the pair with the shortest time-period within the two was selected. If there was evidence of any intervention (either surgical or medical) performed on the patient between the recording of the ECG and the echo in the above selected pair, the ECG-echo pair was discarded and in the absence of any other pairs in the patient record, the patient was not included in the study set.

At the centre, ECG data is captured on Shenzhen Mindray Bio-medical electronics’ BENEHEART R12 devices. A standard sampling rate of 500 Hz with a 10 s interval was used to capture the ECG data. The data are then stored as DICOM images in the inhouse PACS servers for future review and use. We were able to extract ECG from the DICOM images without any loss of data from the site of storage to our study dataset.

Echocardiography is performed using different models of devices manufactured by GE and Philips. This data is stored in the DICOM format on servers which then serves as the basis for the review of the same by experienced cardiac radiologists and cardiologists to create reports for the investigation. The reports are stored on the EMR in a JSON structure as part of an Elastic search implementation on cloud servers. Using random text search keywords, we extracted data and ensured that data association between the ECG and the echocardiographic variables was present using patient identifiers.

Pacemaker patients were retained in the development cohort to ensure the model was exposed to paced ECG morphology during training, given that pacemaker status may not always be known to the system at the point of inference. For the held-out site validation cohort, pacemaker patients were excluded as an exploratory step to examine their influence on performance estimates; the metrics reported correspond to this pacemaker-excluded cohort.

## Data from echocardiography

From the structured echocardiogram table, details regarding Ejection Fraction (EF), Mitral Regurgitation (MR), Tricuspid Regurgitation (TR), Aortic Regurgitation (AR), Pulmonary Artery pressure (PA pressure), Aortic Stenosis (AS), Mitral Stenosis (MS), and Tricuspid Stenosis (TS) were extracted using regular expressions to identify keywords and phrases. These keywords/phrases indicated the exact percentage of EF, the severity of valvular regurgitation/valvular stenosis, and PA pressure from the overall impression validated by the attending cardiologist. An EF of ≤35% was considered as positive for low EF and anything greater than moderate regurgitation was also assigned positive. If the impression on the echocardiogram report indicated ‘mild to moderate’ the higher grade was chosen and was thus assigned ‘positive’ for the purpose of this study. For stenotic lesions, even mild cases were considered pathological. Any PA pressure indicated as ‘increased’ by the cardiologist was bucketed into the positive class. An ‘OR’ operation was then applied to these conditions such that if a patient was positive for one or more of the conditions, the patient was flagged as ‘positive’ for dysfunction, else assigned 0. Thus, the ground truth was a binary variable with each sample having dysfunction or no dysfunction for these eight variables.

## Data from electrocardiography

ECGs were stored in the form of DICOM images, with a ‘YBR_FULL_422’ Photometric Interpretation. Only those ECG images that had all 12 channels stored in a single image were considered for analyses as they constituted the predominant image type, others were excluded from the study. The images were pre-processed by converting them to greyscale, cropping the image to include only the grid containing the ECG trace, and finally resized to (300 × 540) dimensions. The aspect ratio of the image was maintained.

## Ethical considerations

The data engineering team ensured that the personally identifiable information (PII) was masked/removed before the start of modelling. The ECG used had a strip of information above the graph which had information like patient name. This part of the image was cropped out before sending it to the modelling team. Similarly, only the Study ID of the ECG was provided to connect with the corresponding Echocardiography data to ensure that no PII was used for modelling.

## Model implementation and performance

### Model

The models that provided the optimal performance are summarized. The optimal performance was obtained for an ensemble of three models, two InceptionNetV3^14^ models and one ResNet50^15^ model. As the input was a standard ECG Trace Image, and not signals, we were able to leverage the extensive work done on optimizing neural network architectures for computer vision. For all three models, the classification heads were excluded, and a simple global average pooling was done on the output of the last convolution layer after passing it through the rectified linear unit activation function. Finally, a dense layer, with one unit and a sigmoid activation function was added on top of the global average pooled tensor. Thus, each model output is a probability estimate with higher probability indicating risk of having dysfunction (see Supplementary material online, *Figure S1*). The final probability was the arithmetic mean of the three models.

Complete details of model training are provided in the supplemental information.

InceptionNetV3 and ResNet50 were selected on the basis of best empirical performance amongst the CNN architectures evaluated. A systematic comparison against more recent architectures was outside the scope of this work, which was primarily directed at clinical validation of a deployable triage tool rather than architectural benchmarking. The low computational cost of the selected architectures is itself a practical consideration relevant to deployment in resource-constrained settings, and whether newer architectures would confer meaningful additional clinical performance remains an open question for future investigation.

## Model evaluation

All evaluations were done on the test dataset. Model performance was measured by area under the curve for the receiver operator characteristic (ROCAUC) and the precision-recall curves (PRAUC). Sensitivity, specificity, positive predictive value (PPV) and negative predictive value (NPV) were calculated by thresholding the probability distribution. The best thresholds were obtained by calculating the Youden’s Index and F1 score for various thresholds.

Confidence intervals were estimated using a non-parametric bootstrap approach with 1000 iterations, in which the test dataset was repeatedly resampled with replacement. Performance metrics were recalculated for each bootstrap sample, and the 2.5th and 97.5th percentiles of the resulting metric distributions were used to derive the corresponding 95% confidence intervals.

**Calibration:** Model calibration was evaluated on the internal test set to ensure that predicted probabilities were concordant with empirically observed event rates, a prerequisite for valid threshold-based clinical decision-making. Calibration was assessed using a reliability diagram (calibration curve), in which the mean predicted probability within uniform bins was plotted against the observed fraction of positive outcomes, and by the Brier score, a strictly proper scoring rule that jointly captures calibration and discrimination. The distribution of predicted probabilities was examined alongside the reliability diagram to contextualize calibration performance across regions of differing sample density. No post-hoc recalibration was applied.

**Respond-CAM:** With regards to explainability, respond-CAM was applied to generate saliency maps. Respond-CAM is a variant of Class Activation Mapping designed to generate more stable and faithful saliency maps. Instead of using gradients alone (as in Grad-CAM), Respond-CAM weights the feature maps using both the activation values and the gradients, which helps better reflect how strongly each feature map contributes to the model’s output.

**Decision Curve Analysis:** To assess the potential clinical utility of the model, decision curve analysis (DCA) was performed across a range of probability thresholds. Net benefit was calculated by balancing the proportion of true positive detections against the harm associated with false positive classifications. The model’s performance was compared with default strategies of referring all patients for echocardiography or referring none, allowing evaluation of whether the model provides additional clinical value for triaging patients for further imaging.

### External site validation

An independent external validation cohort was assembled from a separate clinical site to evaluate model generalizability. The same inclusion criteria and data extraction procedures used for the development dataset were applied to the external site. Specifically, ECG–echocardiogram pairs were identified using the same temporal linkage criteria, and echocardiographic reports were screened using the same predefined text-search methodology to derive outcome labels. This ensured that case identification and labelling procedures were consistent between the development and external validation datasets.

## Result

51 056 patients’ paired ECG and echocardiograms were identified in the in-house database from the given time-period of which 51 055 patients who were 15 years of age and above were thus included in the study dataset. The distribution of each echocardiographic variable across all the patients is presented in Supplementary material online, *Table S1*. From the total number of patients, we had 53 661 datapoints with 12 channel ECG paired with their echocardiogram selected for the study. The ECG and the echocardiogram were done on the same day.

The overall, no dysfunction vs. dysfunction distribution was 80.76% vs. 19.24% (43 339 vs. 10 322). The mean age distribution of the population was 52.7 ± 12.8 years. The gender distribution of the dataset was approximately 72% males to 28% females. Multiple ECG-echo pairs from the same patient were included in the dataset. Baseline characteristics of the cohort are provided in Supplementary material online, *Table S1*.

The data was split into training (40 796 samples), epoch-monitoring (2148 samples) and testing (10 717 samples) datasets and stratified such that the distribution of each target variable (overall dysfunction) was roughly maintained (see Supplementary material online, *Table S2*). In the testset, 826 patients overlapped with the trainset, and were thus discarded. The testset was thus left with 9788 patients contributing 9873 ECG-echo pairs. The average time between consecutive ECG-echo pairs in the test cohort was 170.4 days.

**Figure 1:**
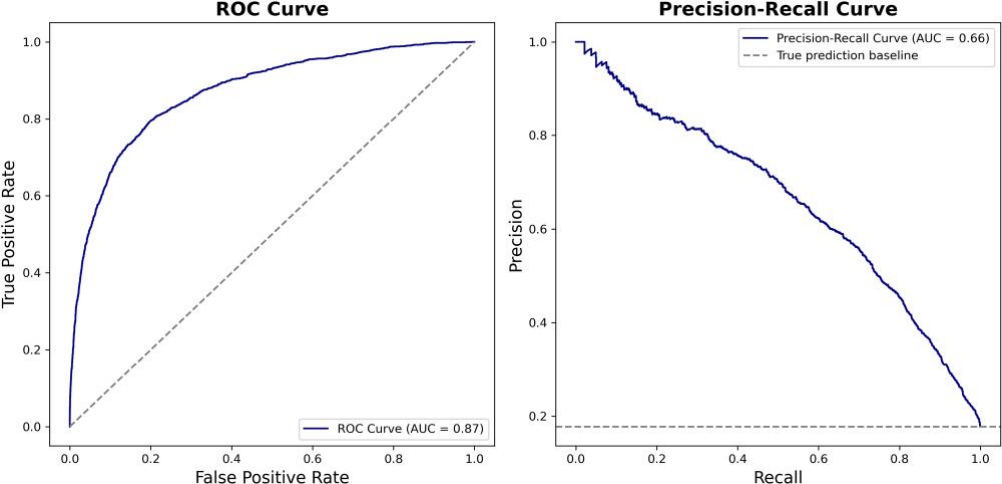
Receiver operating characteristic (ROC) curve and precision-recall (PR) curve comparing the performance of the model. The ROC curve illustrates the trade-off between sensitivity (True Positive Rate) and 1-specificity (False Positive Rate), while the PR curve highlights the precision vs. recall at various threshold levels, particularly useful for imbalanced datasets. The areas under both curves (AUC-ROC and AUC-PR) reflect the model’s ability to distinguish between positive and negative cases.

The global model performance was measured by the ROC and PR-Curves. The AUROC for all the individual models for the binary interpretation of the presence of dysfunction or not was 0.86. The PR AUC for the InceptionNet models were 0.63 and for the ResNet model 0.61. The ensemble model showed better AUC with AUCROC of 0.87 (95% CI: 0.86–0.88) and PRAUC of 0.66 (95% CI: 0.65–0.69) (*Figure 1*).

**Figure 2:**
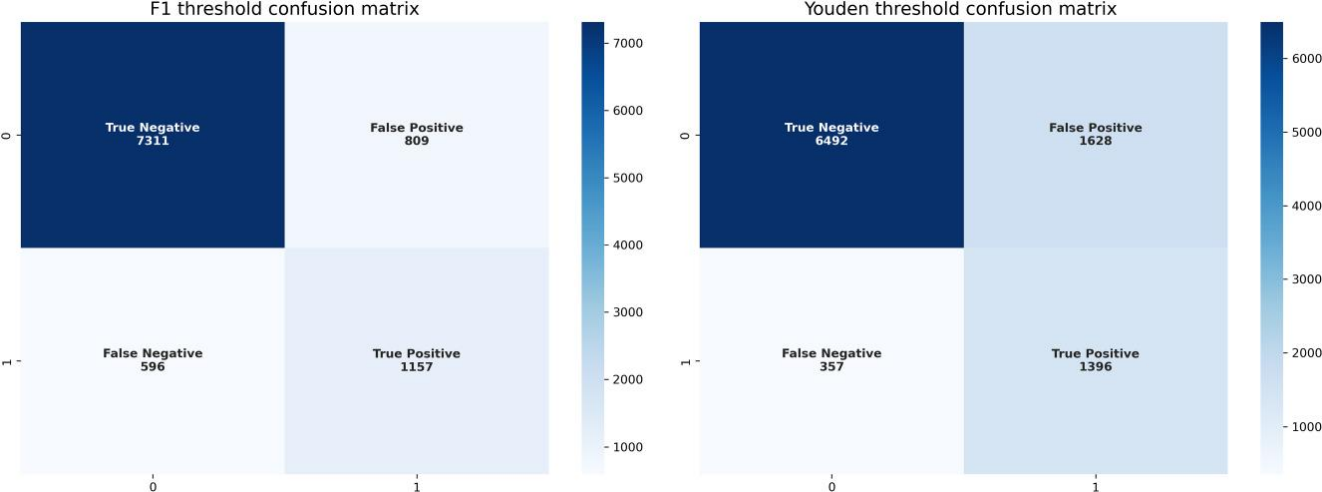
Confusion matrices of the internal test data illustrating performance at both thresholds.

The optimum threshold for the F1 score was 0.40 and optimum threshold based on maximizing the Youden index was 0.27. Based on these thresholds, the output probabilities were binarized and their performance measured. The metrics for each threshold is given in *Table 1*. Their corresponding confusion matrices are surmised in *Figure 2*.

**Table 1:** Diagnostic metrics enumerating the model performance at the F1 score threshold and Youden threshold on the internal test set

METRIC (%)	F1 Threshold (0.40)	CI** (95%)	Youden Threshold (0.27)	CI (95%)
**SENSITIVITY**	0.66	0.65–0.70	0.80	0.79–0.82
**SPECIFICITY**	0.90	0.88–0.91	0.80	0.79–0.80
PPV ***	0.59	0.57–0.61	0.46	0.46–0.50
**NPV ^#^**	0.92	0.92–0.93	0.95	0.94–0.95

Model performance on the testset based on age and gender sub-cohorts are provided in *Table 3*.

*Table 2* enumerates the sensitivity of the ensemble to identify individual echocardiography findings for both the F1 threshold and the Youden threshold. When calculating the sensitivity for a specific condition, it is essential to recognize that patients may simultaneously exhibit other conditions as well.

**Table 2:** Summary of the percentage of individual conditions the model was able to capture on the internal test data

Echocardiographical finding	F1 threshold sensitivity	95% CI	Youden threshold sensitivity	95% CI	*N*, % of testset (*n* = 9873)
**MR**	0.70	0.67–0.73	0.82	0.79–0.84	761, 7.7
**TR**	0.76	0.72–0.80	0.85	0.82–0.89	489, 5.0
**AR**	0.62	0.56–0.68	0.73	0.68–0.78	275, 2.8
**MS**	0.69	0.61–0.76	0.83	0.76–0.89	144, 1.5
**TS**	*0*.*75*	*0.25–1.00*	*0*.*75*	*0.25–1.00*	*4, 0.04*
**AS**	0.63	0.56–0.70	0.72	0.65–0.79	177, 1.8
**Increased PAP**	0.83	0.79–0.86	0.91	0.88–0.93	501, 5.1
**EF ≤35%**	0.86	0.84–0.88	0.94	0.92–0.96	679, 6.9

**Figure 3:**
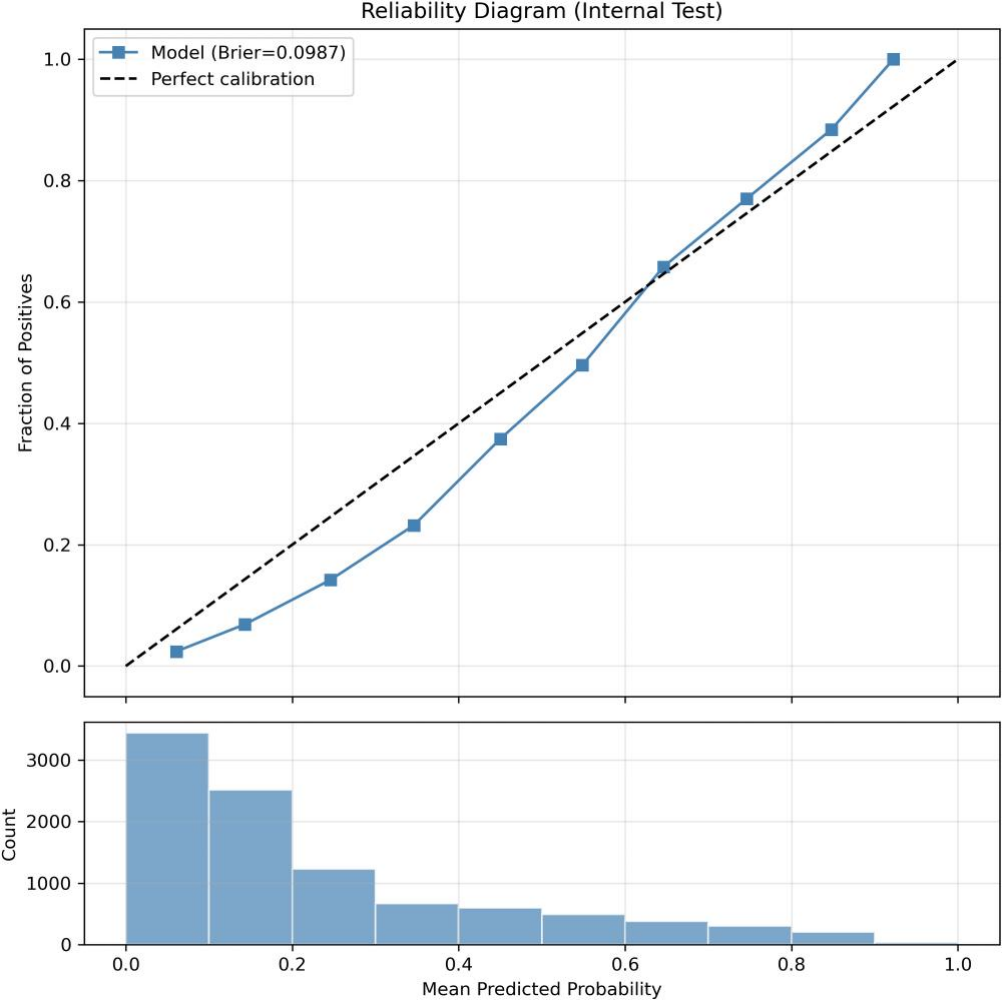
Upper panel: the calibration curve (blue line) plots the mean predicted probability against the observed fraction of positive outcomes (dysfunction) within ten uniform bins. The dashed diagonal represents perfect calibration. The model demonstrates close agreement with the line of perfect calibration across the full prediction range, with a modest degree of overconfidence in the low-to-mid probability region (0.0–0.55) and accurate calibration above this range. The Brier score was 0.099, indicating strong overall probabilistic accuracy relative to a naïve reference model. Lower panel: Histogram of predicted probabilities, illustrating that most predictions concentrate in the 0.0–0.20 range.

**Model calibration**: Model calibration was assessed on the internal test set using a reliability diagram and the Brier score. The model achieved a Brier score of 0.099, comparing favourably against the naïve reference of prevalence × (1 − prevalence) and consistent with strong probabilistic accuracy in clinical prediction modelling. The reliability diagram demonstrated close agreement between predicted probabilities and observed event rates across the full prediction range, with no systematic or clinically meaningful departure from the line of perfect calibration. A marginal degree of overconfidence was observed in the low-to-mid probability range (0.0–0.55), where the calibration curve lay slightly below the diagonal; however, even within this region, which encompasses the majority of predictions, as confirmed by the distribution of predicted probabilities, deviations remained modest. In the mid-range (∼0.55–0.65), the calibration curve intersected the diagonal, reflecting accurate calibration, while a marginal tendency toward under confidence was noted at higher predicted probabilities (0.65–1.0), where sample density was limited (*Figure 3*). Taken together, these findings indicate that the model is well calibrated, that the rank ordering of predicted risk is preserved, and that the threshold-based clinical decisions and DCA derived from model outputs are not materially undermined by miscalibration.

**Figure 4:**
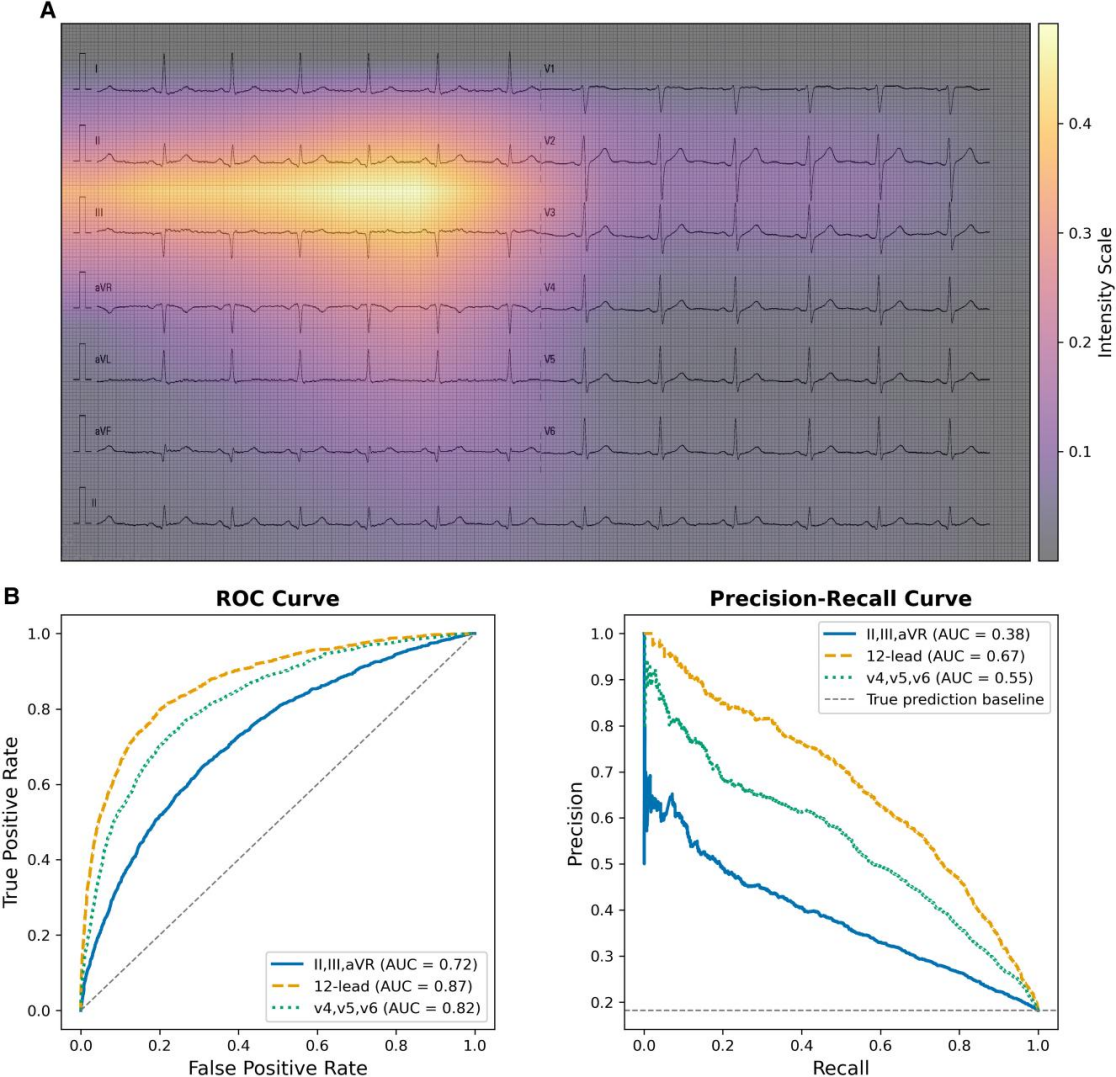
*A*) the average region of interest of the ensemble as generated by the respond-CAM method, superimposed on a sample ECG. *B*) The ROC and PR-AUC curves show that the model performed better with v4, v5, v6 leads when compared with II, III and AvR.

**Saliency maps**: The most common type of trace image format in the dataset was the 2 × 6 matrix with the rhythm strip at the bottom of the image. The respond-CAM^16^ for each of the models in the ensemble was done with the heatmaps generated for all the images. The arithmetic mean of the all the heatmaps for each model was then generated to observe any overall regions of interest on the image that might drive model predictions (for both classes). *Figure 4* shows the average respond-CAMs generated by each model. It was observed that across all models, leads II, III and aVR seemed to be the important regions that drove model predictions. To test this, trace images (2 × 6 with Rhythm strip format) from the test set masking all leads except II, III and avR were first input to one of the InceptionNet models. The trace images were then masked such that only leads v4, v5, and v6 were used as input to the model. The model performance was then compared by plotting the ROC and PR-curves. It was observed that the v4, v5, v6 combination outperformed the II, III and aVR combination (*Figure 4*). This is suggestive that trying to use GRAD-CAM to find ‘leads of interest’ in this method appears to be unfeasible.

MR and low EF were the two most common cardiac dysfunctions observed in this cohort. If cases of low EF are removed from the test cohort, the sensitivity of the model (using the Youden threshold) reduces to 0.71 (95% CI: 0.69–0.74) in capturing the rest of the conditions. Similarly, if MR is removed from the cohort, we observe a minimal/no change in the sensitivity of the model—0.78 (95% CI: 0.77−0.82).

**External site data validation:** ECG-echocardiogram pairs of 21 658 patients were identified and extracted from another hospital within the same group but from a different geographical location (about 1870 km away catering to a different cohort) from January 2022 to March 2024. Corrupted data was excluded followed by excluding those patients who had a pacemaker implant. Totally 20 053 patients were selected for further validation. The cohort included repeated ECG–echo pairs per patient, with an average interval of 125 days between consecutive ECGs and corresponding echocardiograms. The age distribution of the cohort was 55.26 (±13.98) and the gender distribution was ∼2.3 males to every female. From all the patients 22 055 ECG-echocardiogram pairs were identified, and the ground truth generation (overall dysfunction) and ECG image preprocessing was performed. The distribution of dysfunction vs. non-dysfunction was 16.35% vs. 83.65%. The distribution of individual dysfunctions is provided in *Table 3* of the supplemental information. The ECG images were then fed to the model, and the following results were observed. The ROC-AUC was 0.84 and PR-AUC was 0.50. The sensitivity, specificity, PPV and NPV were 0.62, 0.87, 0.49 and 0.92 respectively for the F1 threshold. For the Youden Index threshold the same metrics were 0.82, 0.73, 0.37 and 0.95.

**Table 3:** Test-set sub-cohort analysis (Youden Index threshold). All metrics reported on held-out test set

Subgroup	N	N pos.	Prev %	ROC AUC	Sensitivity	Specificity	PPV	NPV	F1
Gender									
Female	2809	548	19.5	**0.862**	**0.807**	**0.780**	**0.470**	**0.943**	**0.594**
Male	7064	1205	17.1	**0.873**	**0.792**	**0.807**	**0.458**	**0.950**	**0.580**
**Age**									
< 50	3944	543	13.8	**0.907**	**0.794**	**0.881**	**0.516**	**0.964**	**0.626**
50–65	4050	746	18.4	**0.856**	**0.786**	**0.777**	**0.443**	**0.941**	**0.566**
> 65	1879	464	24.7	**0.809**	**0.817**	**0.657**	**0.438**	**0.916**	**0.570**

Performance at the external site was comparable with and without pacemaker patients included (see Supplementary material online, *Table S5*), with sensitivity and NPV remaining unchanged at 0.82 and 0.95 respectively at the Youden threshold, confirming that the development-validation asymmetry in pacemaker inclusion did not materially influence the reported metrics.

## Discussion

### Principal findings and clinical implications

This study presents a deep learning model that predicts major echocardiographic abnormalities using 12-lead ECG images in DICOM format. The model was trained and tested in an Indian outpatient cohort, focusing on eight key dysfunctions: AS, MS, TS, AR, MR, TR, elevated PAP, and EF ≤ 35%. The primary aim was to develop a triage tool to reduce unnecessary echocardiograms in adults while maintaining high NPV.

The model achieved an AUROC of 0.87 and a PR-AUC of 0.66 in internal testing, with an NPV of 0.95 at the Youden threshold. The external validation across a geographically distinct cohort yielded similar NPV (0.95) but slightly lower AUROC (0.84) and PR-AUC (0.50). These findings support the use of this AI tool as a screening mechanism to rule out significant cardiac dysfunction, especially in resource-limited settings.

### Clinical trade-off and false negative risk

The present model is intended as a triage and prioritization tool to assist in the allocation of echocardiography resources in high-volume or resource-constrained settings, and should not be interpreted as a standalone rule-out test for structural heart disease. As with established cardiac investigations such as exercise stress testing, its clinical utility is best realized within structured diagnostic pathways and in conjunction with the ordering clinician's judgement. At the Youden threshold, in the external validation cohort, the model achieved a sensitivity of 0.82, implying that ∼1 in 5 patients with true dysfunction may not be flagged. Analysis of false negative cases reveals that the majority of missed pathology occurred in patients with mild-to-moderate valvular lesions or borderline reductions in ejection fraction (95% of all false negatives), while a smaller proportion (5%) involved more significant abnormalities (see Supplementary material online, *Table S4*). These findings contextualize the nature of the missed diagnoses and underscore that the risk of failing to detect severe, immediately actionable disease is substantially lower than the aggregate false negative rate suggests. The choice of operating threshold should be context-dependent: higher sensitivity thresholds are preferable in settings where minimizing missed pathology is paramount, whereas more conservative thresholds may be appropriate where the primary goal is resource prioritization under adequate clinical oversight. These considerations collectively inform how the model should be deployed, and reinforce that clinical judgement remains central to the final decision to refer for echocardiography.

Age-stratified analyses reveal a clinically important performance gradient that warrants explicit discussion. The AUROC declines from 0.91 in patients under 50 years to 0.81 in those who are 65 years and above, with specificity falling from 0.881 to 0.657 and PPV from 0.516 to 0.438, the latter implying that the majority of positive screens in the oldest and most commonly referred subgroup would not be confirmed on echocardiography. We hypothesize, and demonstrate empirically, that this finding is substantially attributable to the application of a globally derived decision threshold (0.27) to a subgroup whose predicted probability distribution is systematically shifted upward by age-related ECG confounders and higher disease prevalence. When the Youden threshold is re-derived independently within the ≥65 subgroup, the optimal value rises to 0.362, a 34% above the global threshold. Applying this age-specific threshold yielded a modest reduction in sensitivity (0.81 to 0.73), but recovers specificity to 0.774 and improves PPV to 0.517, while the NPV remains clinically acceptable at 0.900. These findings suggest that age-stratified threshold calibration represents a tractable and clinically grounded approach to improving triage precision in elderly patients. Prospective validation of age-specific operating thresholds in independent cohorts is a priority for future work, and clinical deployment in this subgroup should, in the interim, be undertaken with heightened clinical oversight and pre-test probability assessment by the referring clinician.

### Comparison with prior work

Our model distinguishes itself in terms of input format, pathology breadth, and deployment context. Prior work by Elias *et al*.^17^ Cohen-Shelly *et al*.^18^ Vaid *et al*.^19^ and Cinq-Mars *et al*.^20^ used structured waveform data and targeted single or narrow groups of valvular pathologies (e.g. AS, MR). In contrast, our model operates directly on ECG image files (DICOM), offering broader applicability in real-world environments where waveform data may not be available. Additionally, our model simultaneously screens for a wider spectrum of structural dysfunctions. The value of the study is replicated in the meta-analysis by Singh *et al*.^21^ who also demonstrated the value of using machine learning techniques for the diagnosis and screening of valvular heart disease, albeit in an individual lesion by lesion manner, and not in the combined manner as in our study.

While Cinq-Mars *et al*.^20^ reported AUROCs ranging from 0.74 to 0.86 for detecting valvular disease using data collected within a 14-day window, our study minimized temporal variability by ensuring same-day ECG-echocardiogram pairing. This design choice likely improved labelling fidelity and reduced misclassification due to clinical evolution. Notably, our model’s generalizability held in a lower-prevalence population, further supporting its scalability.

While prior work^22^ has demonstrated the feasibility of detecting structural heart disease from digitally sampled ECG waveforms, our approach differs in several important ways. First, our model operates directly on standard 12-lead ECG images, rather than raw waveform signals. This enables deployment in settings where waveform data are not stored or accessible, which is common in many healthcare systems including India. Second, the model requires no additional structured inputs beyond the ECG itself (age; sex; atrial rate; ventricular rate; pulmonary regurgitation interval; Q wave, R wave and S wave (QRS) duration; and corrected Q wave-to-T wave interval), simplifying integration into clinical workflows. Third, an image-based pipeline avoids dependence on vendor-specific ECG management systems and allows inference from scanned ECGs, PDFs, or photographed ECGs, improving portability across institutions. Finally, our work specifically evaluates the model as a triage tool for echocardiography referral, addressing the clinical problem of optimizing echocardiography utilization.

Prior work in diagnosing valvular heart disease using chest x-rays have also been done^23^. This work mirrors the work done by them in the manner that it detects abnormalities arising on the electrocardiogram from a multitude of pathological cardiac conditions thereby creating a test to diagnose a panel of cardiac diseases. The main difference in the two being that the input data in that study was chest x-ray and in ours was images of the ECG.

We also use the images as input since it is the most easily available form of an electrocardiogram in our setting. Unlike the work, by Tison *et al*.^24^ who segmented the electrocardiogram into its component waveforms and intervals, this method allows for easy data use as input into the algorithm.

Oikonomou *et al*.^25^ have also shown that following up patients classed initially as false positives by ECG-AI showed significant increase in F1 scores when this cohort was followed up longitudinally. Future work will include a longitudinal evaluation of the false-positive cohort within the Indian population to determine whether the model captures preclinical signals that are not yet detectable on imaging.

### Decision curve analysis: clinical utility

**Figure 5:**
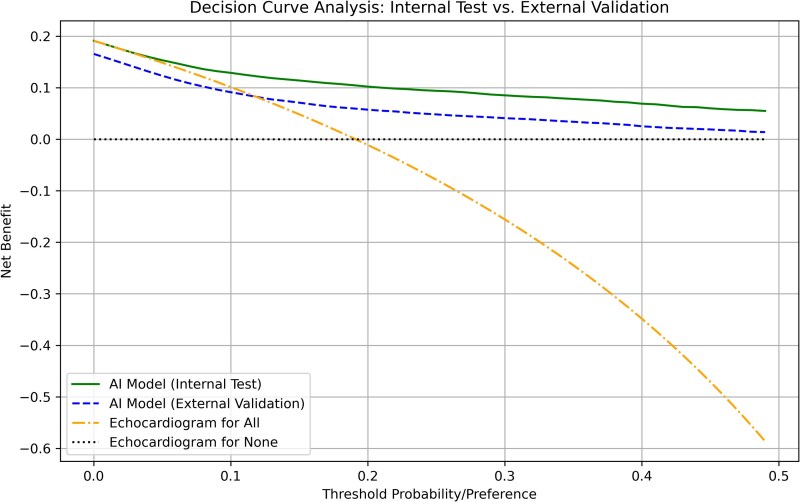
The DCA of the model performance on both internal and external data between the 0 to 0.5 probability thresholds. This range was chosen based on the Youden (0.27) and F1 (0.40) thresholds calculated on the internal set.

To assess real-world decision-making impact, we performed DCA (*Figure 5*). The AI model outperformed both extremes ‘echocardiogram for all’ and ‘echocardiogram for none’ across a wide range of threshold probabilities (0.05 to 0.45). The internal test set demonstrated the greatest net benefit, with the external validation cohort also maintaining clinically meaningful utility. These results support the algorithm’s role as an effective triage tool that could reduce unnecessary testing and streamline resource allocation.

It is important to contextualize the ‘echo for all’ comparator arm within this study's specific clinical setting. Unlike population-level screening studies where this strategy implies indiscriminate referral, every patient in our cohort had already undergone clinical evaluation by a physician at a tertiary cardiac centre prior to echocardiography referral. The ‘echo for all’ arm therefore operationalises the current standard of informed clinical practice, integrating history, examination, and clinical judgement rather than a simple or uninformed baseline. The disease prevalence of ∼17–20% observed in the test cohort reflects this prior clinical enrichment and is embedded in the net benefit computation. The AI model's incremental net benefit above this arm should therefore be interpreted as value added over an already clinically filtered referral standard, not over an unenriched population. Future prospective studies applying this model in unselected outpatient populations, with direct comparison against structured clinical scoring tools, will be necessary to characterize its utility earlier in the triage pathway.

### Population, prevalence, and external-site validity

Our dataset represents a multi-ethnic, tertiary referral population with a disease burden of 19.24%. External validation in a separate Indian centre with similar demographics confirmed the model’s robustness despite slight shifts in disease prevalence (16.35%). These findings suggest the algorithm could generalize well beyond the initial study site, though prospective validation remains essential.

The economic implications include reduction in the downstream diagnostic costs by avoiding unnecessary echocardiograms, with minimal risk of missing pathology. This potential aligns with healthcare efficiency goals in high-volume, resource-constrained settings.

### Robustness to pathology composition

Sensitivity analyses showed that the model's performance remained stable even when the most common pathologies (e.g. MR and low EF) were removed from the test cohort. This suggests the algorithm's ability to generalize across less prevalent dysfunctions, highlighting its utility in heterogeneous clinical populations. To our knowledge, this type of internal robustness analysis has not been reported in similar ECG-AI studies.

### Limitations in explainability: negative findings

Attempts to improve model interpretability using GRAD-CAM were inconclusive. Although heatmaps frequently emphasized leads II, III, and aVR, empirical testing revealed that lead sets V4–V6 provided superior predictive performance. This disconnect suggests that class activation maps derived from ECG images may not reliably indicate physiologically relevant features. These negative findings caution against over-interpreting saliency maps and underscore the need for more clinically grounded explainability tools tailored to image-based ECG inputs. Unlike in the paper by Bernard *et al*.^26^ we were unable to find any specific pheno groups that would help improve our understanding on how the detection of these conditions on the ECG could be linked both associatively and positively to the underlying cardiac disease.

### Study limitations

The composite outcome label includes tricuspid stenosis (TS), which was represented by only 4 cases in the internal test set (0.04% of the cohort). At this sample size, the condition-specific sensitivity estimate is statistically uninformative yielding a 95% confidence interval of 0.25–1.00 that spans the full clinically plausible range. No reliable conclusions can be drawn about model performance in detecting isolated TS. This reflects the rarity of rheumatic tricuspid stenosis in the study population rather than a sampling deficiency, but prospective studies recruiting from populations with higher rheumatic heart disease burden would be required to evaluate model performance on this condition adequately.

A further limitation specific to the image-based nature of this approach is that all ECGs in both the development and external validation cohorts were acquired on a single device type (Mindray BENEHEART R12) and therefore share a common visual layout, grid formatting, and trace rendering convention. Generalizability to ECG images generated by other vendors, where page layout, scaling, font, and DICOM image formatting may differ substantially, has not been evaluated in this current study. Prospective validation across multiple device types and image formats will be a necessary step before broader clinical deployment, and image standardization or vendor-agnostic preprocessing pipelines may be required to ensure consistent model performance across heterogeneous acquisition environments.

This study is limited by its retrospective design and single-country origin. The model was trained on an Indian population and may not generalize to populations with different disease spectra or healthcare infrastructures. While ‘external-site’ validation was performed, both sites operate within the same institutional network, sharing clinical protocols, EMR systems, and ECG acquisition devices. This limits the degree of true independence of the validation.

The age-stratified threshold analysis findings suggest that the observed reduction in PPV in older patients is influenced, in part, by the use of a globally derived threshold that does not fully account for subgroup-specific probability distributions. In older patients, the current model may be more appropriately used as a prioritization tool rather than a filtering mechanism, given the higher false-positive rate.

Future prospective studies are needed to confirm utility and safety in real-time workflows. Legal, regulatory, and cost-effectiveness analyses also remain outside the scope of this study but will be critical for deployment.

## Conclusion

Prior ECG-AI studies have demonstrated the feasibility of detecting structural cardiac abnormalities, pre-dominantly in Western populations. This study replicates and extends those findings to a large Indian tertiary care cohort, while reframing the clinical question: rather than screening an unselected population for undetected disease, the model triages patients referred for echocardiography, identifying those at sufficiently low risk to safely defer imaging. The model achieves an AUROC of 0.87 internally and 0.84 on external site validation, with a negative predictive value of 0.94 to 0.95 preserved across both cohorts, supporting its role in reducing unnecessary echocardiographic investigations without compromising the detection of clinically significant disease.

The tool is not intended as a replacement for clinical judgement or as a standalone rule-out test. Its value lies in augmenting the referring clinician's assessment in high-volume, resource-constrained settings where echocardiography demand routinely exceeds capacity. Used in this way, the model potentially offers a pragmatic, scalable, and low-cost adjunct to existing triage workflows, requiring only the ECG image that is already routinely acquired.

Several important limitations must be resolved before clinical deployment, including prospective validation in an independent health system, evaluation across ECG devices from multiple vendors, and recalibration of operating thresholds in elderly populations. These represent clear and tractable directions for future work.

If these conditions are met, AI-augmented ECG triage has the potential to meaningfully reduce unnecessary echocardiographic investigations, improve the allocation of a scarce specialist resource, and ultimately shorten the diagnostic pathway for patients with genuine cardiac disease.

## Supplementary Material

ztag082_Supplementary_Data

## Data Availability

The data underlying this article cannot be shared publicly due to patient privacy considerations and institutional data governance policies. All patient data were collected retrospectively from electronic medical records of Narayana Health, Bengaluru, and the Rabindranath Tagore International Institute of Cardiac Sciences, Kolkata, under ethics committee approval (NHRTIICSEC/INV/Non-Reg/2023/012). The data remain subject to institutional restrictions governing their external distribution. The data will be shared on reasonable request to the corresponding author (Dr. Abhyuday Kumara Swamy; abhyuday.kumaraswamy@narayanahealth.org), subject to institutional approval and the execution of an appropriate data sharing agreement.
