# Supplementary material for: AI-augmented ECG for pre-echocardiography triage: a tool to optimize cardiac imaging utilization
Source: Eur Heart J Digit Health. 2026 Jun 12;7(5):ztag082. doi: 10.1093/ehjdh/ztag082 (PMC13267140; doi:10.1093/ehjdh/ztag082)
Supplement: ztag082_Supplementary_Data [file ztag082_supplementary_data.docx]

**SUPPLEMENTAL INFORMATION:**

MODEL INITIALIZATION AND INPUT**:**

- The models were initialised with **random weights** (weights=None), without ImageNet transfer learning, to allow training from domain-specific ECG image data without pre-trained feature bias
- The input layer accepted **ECG images of fixed dimensions (300 × 540 pixels, single channel / grayscale)**, standardising all inputs prior to inference
- A **Global Average Pooling** layer followed the backbone, reducing spatial feature maps to a single feature vector and mitigating overfitting compared to fully connected flattening
- A single **Dense output neuron with sigmoid activation** produced a probability score between 0 and 1, corresponding to the likelihood of any significant cardiac dysfunction (composite outcome)

MODEL ARCHITECTURE:


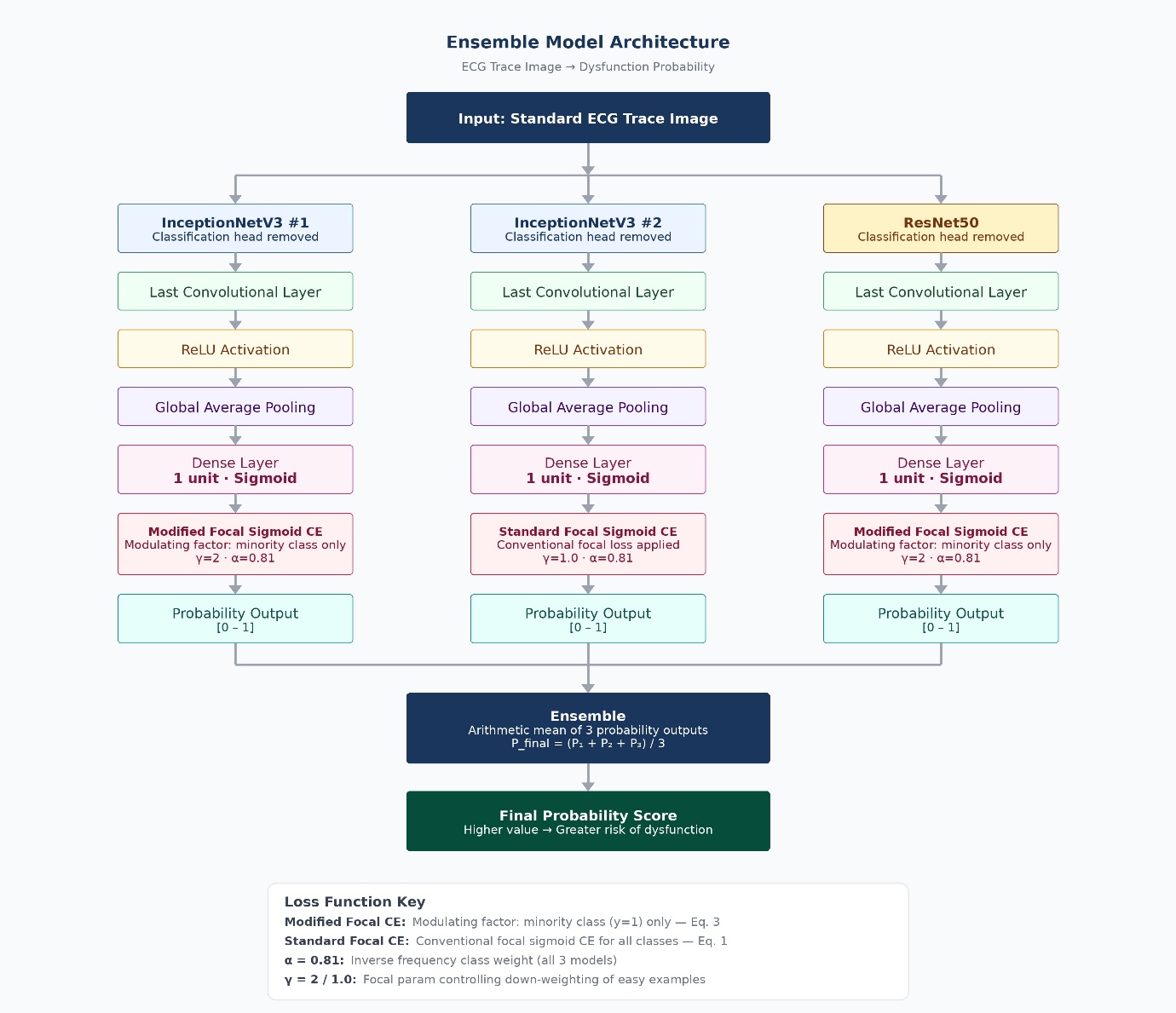


**Figure 1:** Overall model ensemble showing final probability score generated by calculating the arithmetic mean of the individual model probability outputs.

LOSS FUNCTION:

A modified Focal Sigmoid Cross Entropy loss ^1^ was used as the cost function for two of the models of the ensemble. Since this allows the model to focus not only on the imbalanced class in a dataset, but also penalizes the model more for mistakes on hard examples, which are examples that are difficult to classify correctly, irrespective of the class; we found that by modifying the conventional focal sigmoid cross entropy loss by applying the ‘modulating factor/focal parameter’ only to the class of interest (samples that had dysfunction), the models performed better in many of the cases. The best performing models were ensembled and of the three models in the ensemble, the modified loss function was applied for two. The conventional focal sigmoid loss was employed for the other.

Focal sigmoid cross entropy loss is given by –

𝐹𝐿(𝑝𝑡)=−𝛼(1−𝑝𝑡)^𝛾^ log(𝑝𝑡) [Eq. 1]

Where:

- 𝑝𝑡 = (*p*) if y = 1 or (1 – *p*) otherwise. *p* ∈ [0, 1] and y = ground truth.
- *γ* is the hyperparameter the regulates the rate at which the easy classifications are down weighted.
- 𝛼 is the inverse frequency class-weighting.

𝛼(1−𝑝𝑡)^𝛾^  is the modulating factor. For one of the InceptionNet models and the ResNet models, we modified eq. (1) such that the modulating factor was applied only to the minority class (i.e., class 1, the class of interest, dysfunction).

$focal weight = \alpha\cdot\left( 1.0 - p_{i} \right)^{\gamma}$ [ Eq. 2]

$modified loss=-\sum_{i=1}^{n} (focal weight \cdot y_{i} \cdot log(p_{i}) + (1.0 - y_{i}) \cdot log(1.0 - p_{i}))$ [ Eq. 3]

Where:

- y = ground truth, p = predictions and n = batch size.

We found that empirically, models often performed better with this modification, (Eq. 3) rather than just applying the conventional focal sigmoid cross entropy (Eq. 1). We found that setting gamma=2 and alpha=0.81 (inverse frequency weight) gave us the best results. For the remaining InceptionNet model, the usual focal sigmoid loss function (Eq. 1) was used (gamma=1.0; alpha=0.81).

OPTIMISER:

The **Adam optimiser** was used with default hyperparameters, providing adaptive learning rate scheduling appropriate for deep network training on medical imaging data

TRAINING OBJECTIVE AND EVALUTATION METRIC:

- The primary training metric was **AUC (Area Under the ROC Curve)**, consistent with the binary classification objective and reflective of discriminative performance across all classification thresholds — appropriate for a triage screening tool where the operating threshold may be adjusted contextually

REGULARISATION & OVERFITTING MITIGATION:

- **Early stopping** was applied monitoring epoch-monitoring set loss, with a patience of 3 epochs and no minimum delta, halting training upon failure to improve generalisation performance
- **Model checkpointing** saved only the best-performing epoch (by validation loss), ensuring the final model reflected peak generalisation rather than terminal training state

MODEL SELECTION AND VERSIONING:

- The saved model (_v1.h5) was identified by its loss function configuration and outcome label, providing **transparent versioning** traceable to the composite outcome definition (valvular disease, elevated pulmonary artery pressure, EF ≤35%)

TABLE 1: Baseline characteristics of the development cohort.

| Characteristic | Value | Missing |
| --- | --- | --- |
| Diabetes mellitus, n (%) | 17137 (33.6%) | 0 (0.0%) |
| Hypertension, n (%) | 21271 (41.7%) | 0 (0.0%) |
| Cerebrovascular accidents, n (%) | 751 (1.5%) | 0 (0.0%) |
| Myocardial Infarction, n (%) | 581 (1.1%) | 0 (0.0%) |
| Hypercholesterolemia, n (%) | 5756 (11.3%) | 25128 (49.2%) |
| Serum Creatinine (mg/dL), mean ± SD | 0.92 ± 0.59 | 21208 (41.5%) |
| Age (years), mean ± SD | 52.59 ± 12.78 | 0 (0.0%) |
| Body mass index (kg/m²), mean ± SD | 25.86 ± 4.79 | 47698 (93.4%) |
| Sex, n (%) |  | 0 (0.0%) |
| Male | 36596 (71.7%) | 1 (<0.001%) |
| Female | 14458 (28.3%) |  |
| Age Groups, n (%) |  | 0 (0.0%) |
| <40 | 8449 (16.5%) |  |
| 40–49 | 11700 (22.9%) |  |
| 50–59 | 14416 (28.2%) |  |
| 60–69 | 12129 (23.8%) |  |
| 70–79 | 3936 (7.7%) |  |
| ≥80 | 425 (0.8%) |  |

TABLE 2: Train, epoch-monitoring and testset split.

| VARIABLE | PRESENT  n (%) | ABSENT  n (%) | TRAINSET  PRESENT/ABSENT  (%) | EPOCH-MONITORING SET PRESENT/ABSENT  (%) | TESTSET  PRESENT/ABSENT  (%) |
| --- | --- | --- | --- | --- | --- |
| MR | 4396 (8.19) | 49,265 (91.81) | 8.17/91.83 | 8.29/91.71 | 7.7/92.3 |
| TR | 2946 (5.49) | 50,715 (94.51) | 5.52/94.48 | 5.68/94.32 | 5.0/95.0 |
| AR | 1780 (3.32) | 51,881 (96.68) | 3.42/96.58 | 3.35/96.65 | 2.8/97.2 |
| Increased PASP | 3051 (5.69) | 50,610 (94.31) | 5.72/94.28 | 5.82/94.18 | 5.1/94.9 |
| AS | 1082 (2.02) | 52,579 (97.98) | 1.99/98.01 | 2.51/97.49 | 1.8/98.2 |
| TS | 36 (0.07) | 53,625 (99.93) | 0.08/99.92 | 0.05/99.95 | 0.04/99.96 |
| MS | 909 (1.69) | 52,752 (98.31) | 1.72/98.28 | 1.77/98.23 | 1.5/98.5 |
| LOW EF | 3824 (7.13) | 49,837 (92.87) | 7.1/92.9 | 6.47/93.53 | 6.9/93.1 |
| DYSFUNCTION | **10,322 (19.24)** | **43,339 (80.76)** | **19.24/80.76** | **19.88/80.12** | **19.1/80.9** |

*NOTE: The testset column reflects the initial stratified partition (10,717 samples) prior to removal of 826 patients overlapping with the training set. The final analysis test set comprised 9,873 ECG–echo pairs; its dysfunction prevalence is reported in Table 3 of the main manuscript.*

TABLE 3: Distribution of Dysfunction in the external site validation dataset.

| VARIABLE | PRESENT (n, %) | ABSENT (n, %) |
| --- | --- | --- |
| MR | 558 (2.53) | 21,497 (97.46) |
| TR | 768 (3.48) | 21,287 (96.52) |
| AR | 151 (0.68) | 21,904 (99.32) |
| Increased PASP | 2053 (9.31) | 20,002 (90.69) |
| AS | 60 (0.27) | 21,995 (99.73) |
| TS | 6 (0.03) | 22,049 (99.97) |
| MS | 43 (0.19) | 22,012 (99.81) |
| LOW EF | 1645 (7.46) | 20,410 (92.54) |
| DYSFUNCTION | **3606 (16.35)** | **18,449 (83.65)** |

TABLE 4: False negative analysis done on the internal test-set.

| **False Negative Analysis by Condition and Severity Category** | | | |
| --- | --- | --- | --- |
| **Condition** | **Total FN** | **Non-Severe FN, n (%)** | **Severe FN, n (%)** |
| Mitral Stenosis (MS) | 27 | 26 (96%) | 1 (4%) |
| Aortic Stenosis (AS) | 49 | 38 (78%) | 11 (22%) |
| Tricuspid Stenosis (TS) | 1 | 1 (100%) | 0 (0%) |
| Mitral Regurgitation (MR) | 135 | 133 (99%) | 2 (1%) |
| Aortic Regurgitation (AR) | 74 | 72 (97%) | 1 (1%) |
| Tricuspid Regurgitation (TR) | 71 | 71 (100%) | 0 (0%) |
| Reduced EF | 42 | 38 (90%) | 4 (10%) |
| **Overall (valvular + EF)** | **399** | **379 (95%)** | **19 (5%)** |

*FN = false negative (patients with the given abnormality not flagged by the model at the Youden threshold on external validation).*

*Non-Severe: mild, mild-to-moderate, or moderate grade for valvular lesions; EF ≥30% for reduced ejection fraction.*

*Severe: severe or significant grade for valvular stenosis/regurgitation; EF <30% for reduced ejection fraction.*

*For aortic regurgitation, moderate-to-severe grade is classified as non-severe (n = 4); one AR false negative could not be grade-classified from available data.*

*Pulmonary artery pressure (elevated PAP, n = 45) is excluded from this table as severity grading by ECG-predicted PAP threshold is not applicable in this context.*

*Percentages for individual conditions are calculated as a proportion of the condition-specific false negative total.*

*Overall percentages are calculated as a proportion of the combined false negative total (n = 399*).

Disambiguation of PA pressure not provided as data was collected as increased vs not increased; there were 45 False negatives in this subset out of an overall 501 cases ~9% False negatives.

TABLE 5: External-site model performance on the pacemaker vs non-pacemaker cohort.

| Metric | With Pacemaker (n=24,237 pairs) | Without Pacemaker (n=22,055 pairs) |
| --- | --- | --- |
| Sensitivity | **0.82** | **0.82** |
| Specificity | **0.70** | **0.73** |
| PPV | **0.36** | **0.37** |
| NPV | **0.95** | **0.95** |
| Accuracy | **0.72** | **0.74** |

**REFERENCES:**

1. Lin TY, Goyal P, Girshick R, He K, Dollár P. Focal Loss for Dense Object Detection [Internet]. arXiv; 2018 [cited 2023 Jul 18]. Available from: http://arxiv.org/abs/1708.02002
